# Intensive support for adults with intellectual disability and behaviours that challenge: a survey of provision and service typologies in England

**DOI:** 10.1192/bjo.2020.2

**Published:** 2020-02-11

**Authors:** Angela Hassiotis, Amy Walsh, Jessica Budgett, Isobel Harrison, Rebecca Jones, Nicola Morant, Ken Courtenay, Elisabeth Victoria Crossey, Ian Hall, Renee Romeo, Laurence George Taggart, Peter E. Langdon, Victoria Ratti, Vincent Kirchner, Brynmor Lloyd-Evans

**Affiliations:** Professor, Division of Psychiatry, University College London, UK; Research Assistant, Division of Psychiatry, University College London, UK; Research Assistant, Division of Psychiatry, University College London, UK; Project Manager, Division of Psychiatry, University College London, UK; Statistician, Division of Psychiatry, University College London, UK; Qualitative Researcher, Division of Psychiatry, University College London, UK; Consultant Psychiatrist, Haringey Learning Disability Partnership, Wood Green, London, UK; Consultant Psychiatrist, NHS Lothian, UK; Consultant Psychiatrist, East London NHS Foundation Trust, UK; Health Economist, Kings College London, UK; Assistant Professor, Ulster University, UK; Professor, University of Warwick; and Coventry and Warwickshire Partnership NHS Trust, UK; Project Manager, Division of Psychiatry, University College London, UK; Medical Director, Camden and Islington NHS Foundation Trust, UK; Assistant Professor, Division of Psychiatry, University College London, UK

**Keywords:** Intellectual disabilities, intensive support, behaviour that challenges, adults, ID services

## Abstract

**Background:**

Approximately 18% of adults with intellectual disabilities living in the community display behaviours that challenge. Intensive support teams (ISTs) have been recommended to provide high-quality responsive care aimed at avoiding unnecessary admissions and reducing lengthy in-patient stays.

**Aims:**

To identify and describe the geographical distribution and characteristics of ISTs, and to develop a typology of IST service models in England.

**Method:**

We undertook a national cross-sectional survey of 73 ISTs. A hierarchical cluster analysis was performed based on six prespecified grouping factors (mode of referrals, size of case-load, use of outcome measures, staff composition, hours of operation and setting of service). A simplified form of thematic analysis was used to explore free-text responses.

**Results:**

Cluster analysis identified two models of IST provision: (a) independent and (b) enhanced provision based around a community intellectual disability service. ISTs aspire to adopt person-centred care, mostly use the framework of positive behaviour support for behaviour that challenges, and report concerns about organisational and wider context issues.

**Conclusions:**

This is the first study to examine the delivery of intensive support to people with intellectual disability and behaviour that challenges. A two-cluster model of ISTs was found to have statistical validity and clinical utility. The clinical heterogeneity indicates that further evaluation of these service models is needed to establish their clinical and cost-effectiveness.

The lack of the right local support for people with intellectual disability (also known as learning disability in UK health services) and behaviour that challenges across the lifespan has concerned family carers, policymakers and commissioners for many years. Prevalence of behaviour that challenges for example self-injurious, aggressive and inappropriate sexual behaviour among people with intellectual disability in the UK is currently estimated at 18%.^1,2^ It is established beyond doubt that these individuals are subject to increased rates of admissions to hospital, unnecessary long-term use of psychotropic medication, other restrictive practices and poorer physical health.^3,4^ Failure to manage behaviours that challenge before reaching a crisis point causes distress and burden to families and paid carers. Additionally, the individuals are at a higher risk of in-patient admission, and for one in five individuals such admissions are in facilities more than 100 miles away from home,^5,6^ while the number of hospital admissions of adults with intellectual disability and people with autism who display behaviours that challenge remain largely unchanged.^6,7^

## Specialist support

Successive reports, from as early as 1993^8,9^ have advocated intensive support teams (IST) to effectively manage behaviour that challenges in adults with intellectual disability in the community. Many such teams were developed following deinstitutionalisation to facilitate the delivery of services to people with intellectual disability with or without comorbidities and to provide specialist expertise to meet their healthcare needs.^10^ In several areas, such teams undertook the acute and medium- to long-term management of behaviours that challenge and/or mental ill health in the community. There are a plethora of terms such as ‘peripatetic teams’, ‘assertive outreach teams’ and ‘specialist behaviour teams’ that have been used to describe ISTs.^11,12^ Ayres & Roy^13^ described the development of a multiprofessional outreach team in one area in England that supported adults with intellectual disability and mental ill health or other complex needs to remain in supported housing. The team had a case-load of 26 adults and offered 2700 h of weekly input. Eleven of the patients had an autism spectrum disorder, and 23 of the 26 individuals displayed physical aggression. In the nine years of operation the team discharged eight patients and, at the time of publication, it had accepted another 16 who were at various stages of assessment.

The recently produced consensus national model recommends that integrated specialist multidisciplinary teams should provide intensive 24 h care if needed and that various localities may develop their services in a way that serves the local population adopting best practice.^14^ They are also expected to manage crises and support adults during in-patient care and early discharge.^15^ ISTs should be accessible, focused on maintaining people in their own homes and communities and deliver personalised specialist assessment and behavioural support.^16^ However, the report by the NHS Benchmarking Network for Learning Disabilities shows that only 44% of 54 community intellectual disability services (CIDS) that submitted data had intensive support services and emergency access to services was less commonly available compared with community mental health teams.^17^

To date, there has not been a systematic evaluation of the function, patient outcomes and setting within which ISTs operate. The lack of data on their utility and costs may have hindered the commissioning of such teams in contrast to the generic mental health services where the establishment of home treatment and crisis teams is widely implemented.^4,18^ It is, therefore, essential to develop the evidence for a potentially beneficial policy initiative to guide decisions by service commissioners at a local level and for policymakers and clinicians nationally. We believe that there will be valuable information to also guide development and adaptation of ISTs internationally. In this paper we present findings from the first stage of a national investigation of intensive support teams (IST-ID study).^19^ Our aims were: (a) to examine the geographical distribution and characteristics of ISTs in England; (b) to develop a typology of IST service models and (c) to describe the IST model profiles and contextual characteristics.

## Method

### Study design

We carried out a national survey of ISTs in England. First, a screening questionnaire was developed to identify ISTs with reference to national guidance,^8^ which was sent to all specialist CIDS. The latter were identified through previous research, a web search, clinical commissioning groups (these were launched in England and are responsible for implementing the commissioning roles as set out in the Health and Social Care Act 2012.^20^) and the transforming care partnerships list, which included 48 configurations of CIDS. The screening questionnaire included a list of terms that were used to describe intensive support.

Services that were identified as ISTs, were reviewed independently by members of the project management group to confirm whether they fulfilled IST criteria. Any discrepancies regarding team assignment were resolved by discussion at project management group meetings. Second, we developed a detailed survey for the IST and their managers were invited to take part. To achieve a high completion and return rate we employed a number of strategies such as making both online and hard copy versions of the survey available, sending regular email prompts and contacting late responders directly by telephone. We used Opinio software to manage response data.

The survey consisted of 62 questions divided into 13 sections covering service type and location, opening hours, case-load, staffing, management and funding, service-user characteristics, services provided by the IST, use of outcome measures, referrals, response target times, interventions and assessments, intensity of support and concluding questions. Each section comprised both closed and open-ended questions. The free-text sections addressed the IST's model or philosophy of care, any changes the service might be undergoing or planning, challenges faced by the IST, and priorities for improving the service.

### Ethical approval

The Health Research Authority online tool determined that ethical approval was not required for this phase of the project.^20^

### Statistical analysis

The characteristics of the services were summarised using statistics for the distribution of the data (i.e. count and percentage for categorical measures or median and interquartile range for continuous outcomes which were not normally distributed).

The following areas of interest^21,22^ were utilised to determine grouping variables for a cluster analysis to develop a typology of ISTs:

- setting of service;
- team composition;
- case-load;
- operating hours;
- type of referral permitted;
- use of outcome measures.

All factors were defined as binary measures. Staff were grouped into a number of professional categories (nurse, psychiatrist, psychologist, social or support work and other). The number of professions was calculated for each team. Teams consisting of two or more professional staff categories were defined as multiprofessional. Case-load per team member was calculated as the number of patients per full-time equivalent (FTE) member of staff. A large case-load was defined as 2.5 or more clients per 1 FTE staff member (excluding trainees), which is in line with guidance from the UK Department of Health about mental health crisis teams.^23^

Teams were defined as having extended hours of operation if they offered services for longer than conventional Monday to Friday opening hours (09.00 h to 17.00 h) or any weekend services. Services were defined as allowing self-referral if they accepted referrals directly from new or existing service users eligible to receive specialist intellectual disability services or from their family and paid carers (as opposed to being solely from professional services such as general practitioners, secondary mental health services, police or third-sector organisations). The remaining two grouping factors, setting (whether the team operated as an independent service separate from the CIDS or as enhanced CIDS) and whether the service used outcome measures, were based on direct responses to these two questions in the survey.

A hierarchical cluster analysis was performed using the six grouping variables employing Ward's method^24^ with squared Euclidean distance as the dissimilarity measure. A dendrogram was produced to illustrate the agglomeration of individual services into ever larger clusters. All analyses were carried out using Stata/IC v14.0.

The optimal number of clusters into which to group services was determined by visual inspection of the dendrogram, comparison of dissimilarity measures for different clustering options and a discussion about clinical validity of the proposed models with the project management team.

### Qualitative analysis

Free-text responses from the survey were analysed using a simplified, question-based form of thematic analysis.^25^ Responses to each question were organised into basic topic and opinion-based themes. Two of the authors (A.W. and J.B.) were involved in this process with arbitration by a third (N.M.) to ensure conceptual clarity and consistency. Data extracts were selected to illustrate responses in relation to the identified themes per IST model.

## Results

The screening questionnaire was completed by 236 of the 242 CIDS identified in England (97.5%). Of those, 188 CIDS declared that they referred to 80 ISTs whose managers were sent a web-link or hard copy of the full survey. In total, 73 (91%) returned their questionnaires.

### Service characteristics

**Fig. 1:**
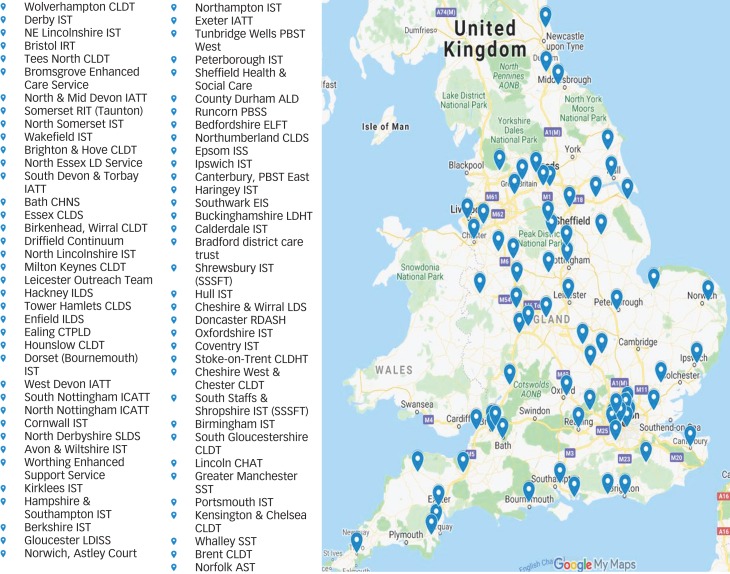
Intensive support teams geographical location map.

Figure 1 shows the geographical distribution of the ISTs and Table 1 summarises key IST service domains. The ISTs were located in Northern England, Midlands and Eastern England, Southern England and London. Most ISTs are funded by the National Health Service (NHS) and the majority employ health and social care staff. Most ISTs operate little to no limitations in accepting individuals relating to behaviours that challenge or mental ill health either in the acute phase or for ongoing care. In total, 16% of teams accept referrals for young people in transition (14- to 17-years-old) and 3% indicated that the IST works across the lifespan. The majority of the IST total workforce are nursing staff with social workers being the second commonest profession. The most frequently provided intervention by ISTs is positive behaviour support but other psychosocial interventions are also reported by the majority of responders.

ALD, Adult Learning Disability; AST, Adult Support Team; CHAT, Community Health Assessment Team; CHNS, Complex Health Needs Service; CLDHT, Community Learning Disability Health Team; CLDT, Community Learning Disability Service; CTPLD, Community Team for People with Learning Disabilities; EIS, Early Intervention Service; ELFT, East London Foundation Trust; IATT, Intensive Assessment and Treatment Team; ICATT, Intensive Community Assessment and Treatment Team; ILDS, Intensive Learning Disability Service; IRT, Intensive Response Team; ISS, Intensive Support Service; IST, Intensive Support Team; LD, Learning Disability; LDHT, Learning Disabilities Health Team; LDISS, Learning Disability Intensive Support Service; LDS, Learning Disability Service; PBSS, Positive Behaviour Support Service; PBST, Positive Behaviour Support Team; RDASH, Rotherham Doncaster and South Humber; RIT, Rapid Intervention Team; SLDS, Specialist Learning Disability Service; SSSFT, South Staffordshire and Shropshire Foundation Trust; SST, Specialist Support Team.

**Table 1:** Intensive support teams (ISTs) characteristics

Characteristic	Values (*n* = 73)
*Location and setting*	
Region, *n* (%)	
Northern England	19 (26)
Midlands and Eastern England	22 (30)
Southern England	23 (32)
London	9 (12)
Funded by NHS, *n* (%)	67 (92)
Funded by local authority, *n* (%)	20 (27)
In social enterprise, *n* (%)	3 (4)
Standalone service, *n* (%)	25 (34)
Length of time in operation, months: median (IQR)	48 (24–96)
*Service users*	
Size of current case-load, median (IQR)	25 (15–50)
Number of service users on at-risk register, median (IQR)	6 (2–15)
Average visit duration, *n* (%)^a^	
30–60 min	25 (35)
60–120 min	43 (60)
>120 min	4 (6)
Extended working hours, *n* (%)	48 (66)
IST operates a duty/crisis line, *n* (%)	38 (52)
Outcome measures used, *n* (%)	55 (75)
*Staffing, training and skills*	
Multiprofessional staff team, *n* (%)	65 (89)
Intellectual (learning) disability nurses, *n* (%)	62 (85)
Clinical psychologists, *n* (%)	57 (78)
Speech and language therapists, *n* (%)	38 (52)
Occupational therapists, *n* (%)	33 (45)
Psychiatrists, *n* (%)	31 (42)
Social workers, *n* (%)	59 (81)
Trainee staff (for example student nurses, trainee associate practitioners), *n* (%)	38 (52)
One or more of team trained as an approved mental health practitioner, *n* (%)	6 (8)
Perceived need for additional training or skills, *n* (%)^b^	50 (68)
*Eligibility and referrals*	
Lower age limit, *n* (%)	
IST accepts adults only (people aged 18 years and above)	59 (81)
IST accepts young people (aged 14–17 years)	12 (16)
No lower age limit	2 (3)
Upper age limit, *n* (%)	
None	71 (97)
IST accepts patients in contact with the criminal justice system, *n* (%)	65 (89)
IST accepts patients experiencing mental health problems, *n* (%)	71 (97)
IST accepts patients with intellectual disability and challenging behaviour who are not in crisis but need support, *n* (%)	64 (88)
IST provides early hospital discharge support, *n* (%)	72 (99)
Self-referrals accepted, *n* (%)	41 (56)
IST accepts referrals without further assessment from trusted assessors, *n* (%)	14 (19)
Target time to respond to referrer, days: median (IQR)	5 (1–14)
Target time to commence assessment, days: median (IQR)	5 (1.5–14)
Target time to complete assessment, days: median (IQR)	7 (3–28)
IST operates a waiting list	7 (10)
*Interventions used*	
Positive behaviour support, *n* (%)	72 (99)
Psychoeducational interventions with service users’ family or paid carers, *n* (%)	68 (94)
Other evidence-based psychosocial therapies (for example anger management, mindfulness, counselling, cognitive–behavioural therapy), *n* (%)	68 (93)

IQR, interquartile range.

a. One team did not answer this question.

b. For example additional professional roles or additional skills such as intervention and prevention strategies.

### Cluster analysis

The cluster analysis included data from 71 ISTs as we were unable to obtain FTEs for two teams. Inspection of the dendrogram and comparison of associated dissimilarity measures indicated that services could be grouped into two main clusters, which were defined as an enhanced provision and an independent model. Enhanced provision ISTs are more likely to provide a broader CIDS function, and longer-term support (more than 6 months), accept self-referrals, have a large case-load and are less likely to use outcome measures (details are shown in Table 2) compared with the independent model.

**Table 2:** Profiles of the two intensive support team models

	Enhanced provision, *n* (%) (*n* = 25)	Independent, *n* (%) (*n* = 46)
*Grouping factors*		
Self-referral permitted, *n* (%)	25 (100)	16 (35)
Large case-load, *n* (%)^a^	23 (92)	15 (33)
Outcome measures used, *n* (%)	13 (52)	41 (89)
Standalone service, *n* (%)	3 (12)	21 (46)
Multiprofessional staff team, *n* (%)	21 (84)	43 (93)
Extended working hours, *n* (%)^b^	16 (64)	31 (67)
*Other service characteristics*		
Working hours, *n* (%)		
Monday to Friday only 7–8 h	9 (36)	15 (33)
Monday to Friday only 8+ hours	9 (36)	8 (17)
Monday to Friday 7–8+ hours and weekends	5 (20)	13 (28)
Monday to Friday 24 h and weekends	2 (8)	10 (22)
Staffing (full-time equivalent), median (IQR)	5.6 (3.6–9.6)	10.2 (6.8–15.0)
*Level of intellectual disability*		
All levels (mild/moderate/severe/profound), *n* (%)	18 (72)	38 (83)
All except profound, *n* (%)	2 (8)	4 (9)
All except mild, *n* (%)	4 (16)	2 (4)
Other combination, *n* (%)	1 (4)	2 (4)
Clients with a neurodevelopmental disorder (%), mean (s.d.)	62.1 (21.5)	51.1 (22.5)
*Frequency of contact with clients*		
Less than once a week, *n* (%)	1 (4)	2 (4)
Once a week, *n* (%)	8 (33)	9 (20)
Twice a week, *n* (%)	5 (21)	6 (13)
Three or more times a week, *n* (%)	6 (25)	14 (30)
Other, *n* (%)	4 (17)	15 (33)
*Duration of contact with clients*		
1–3 months, *n* (%)	0	7 (15)
3–6 months, *n* (%)	7 (29)	20 (33)
6–12 months, *n* (%)	13 (54)	20 (43)
12 months plus, *n* (%)	4 (17)	4 (9)

IQR, interquartile range.

a. Large case-load: ≥2.5 clients per 1 full-time equivalent staff member.

b. Extended hours refer to working outside 09.00 h and 17.00 h.

**Fig. 2:**
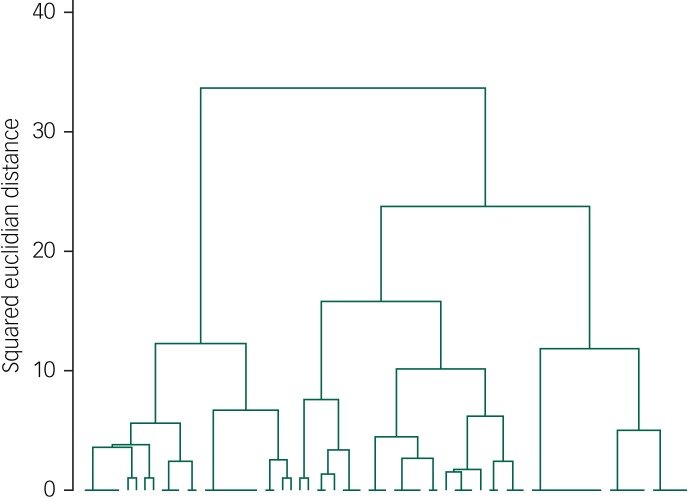
Dendrogram illustrating cluster agglomeration.

There is less evidence that service types are sufficiently distinguished by whether they have a multiprofessional team or operate extended opening hours. Information about the factor clustering is shown in the dendrogram (Fig. 2), which illustrates the agglomeration of individual services into ever larger clusters. Horizontal lines at zero indicate clusters of services that are identical in relation to the grouping factors. Horizontal lines nearer the bottom of the dendrogram represent the merging of clusters that are similar to each other. Horizonal lines nearer the top of the dendrogram represent the merging of more heterogenous clusters, with larger distance values. Long vertical lines indicate that two clusters that are dissimilar to each other are being combined, and suggests that the clusters might represent distinct types of services.

Horizontal lines at zero indicate clusters of services that are identical in relation to the grouping factors. Horizontal lines nearer the bottom of the dendrogram represent the merging of clusters that are similar to each other. Horizonal lines nearer the top of the dendrogram represent the merging of more heterogenous clusters, with larger distance values. Long vertical lines indicate that two clusters that are dissimilar to each other are being combined, and suggests that the clusters might represent distinct types of services.

### Philosophy of care, perceived challenges and priorities for improvement for ISTs

Three survey questions invited free-text comments from respondents on the philosophy of care adopted by ISTs and of the challenges and priorities of the ISTs.

Respondents mentioned the transforming care programme^26^ as a driver behind the care model.

‘The service advocates a PBS [positive behaviour support] approach to supporting service users. The evidence from the team supports that early identification of service user difficulties significantly reduces the likelihood of placement breakdown.’ (Team manager, independent IST)

Others described the care they provided as:

‘Person-centred, holistic ethos, use of positive behaviour support.’ (Team manager, enhanced provision)

Responses indicated that the IST was valued, leading to plans for expansion in its work remit and that employing staff with particular skills or expertise were commented as positive developments.

‘Restructuring of joint LD [learning development] services and appointment of PBS [positive behaviour support] specialist to lead internal and external support staff.’ (Clinical nurse specialist, independent IST)

‘We are going to be enhancing the team and increasing working hours.’ (Lead behaviour nurse, enhanced provision)

The most commonly identified challenges were lack of resources, staff turnover, varying expectations from service providers and quality of residential provider care.

‘Clinical demands high – team have not been fully resourced since its start date. Retention of staff and recruitment problematic. Long periods without team manager in place. Difficult to function as an “Intensive” support team and meet referral needs and manage risks and trust targets/expectations.’ (Team manager, Independent IST)

‘Working with specialist residential providers who do not obviously have the degree of specialism that they advertise in winning support contracts (e.g. little to no specialist training for staff around autism, communication, challenging behaviour and/or mental health), who then become reliant on our team for longer than we are able to lead on all aspects of mental health/behavioural assessment and support’ (Team manager, independent IST)

Priorities included the implementation of national policies, a more flexible approach to providing intensive support outside of working hours and developing reliable liaison with other local agencies in order to improve communication and referral procedures.

‘Improving links with mainstream services and inputting on reasonable adjustments. Improving awareness in out of hours services to avoid hospital admissions out of hours.’ (Team manager, independent IST)

‘addressing the STOMP (overmedication with antipsychotics) agenda, develop service user involvement in the IST work and improving risk management’ (Team manager, enhanced provision)

Clinical pathways were noted as areas requiring improvement for some ISTs, as was enhancement of IST workforce skills through specialist training and adopting evidence-based practice. Staff training in positive behaviour support was identified as an important part of delivering person-centred care.

‘Increase up-skilling of staff teams to reduce ongoing reliance on service e.g. PBS [positive behaviour support] skills etc.’ (Team manager, enhanced provision)

Expanding the IST function to work with an all-age service-user population, particularly those under the age of 18 years, or patients with autism and other population groups such as those with borderline intellectual functioning were identified as potential future additions to ISTs.

## Discussion

The policy report Transforming Care for People with Learning Disabilities: Next Steps^27^ stated that: ‘although a good deal of work has been done to describe what community-based services for people with learning disabilities and/or autism should look like, we have heard from many commissioners a desire for this to be drawn together more clearly into service models and quality standards’. Although there have been previous studies of ‘peripatetic teams’ for adults with intellectual disability and behaviours that challenge,^11,12,17^ this is the first study to our knowledge that has investigated the geographical distribution, characteristics and models of IST care in England. A cluster analysis using six grouping variables resulted in two IST models (independent and enhanced provision) with clinical utility as interpreted by the coapplicant clinicians project management group members. Distinguishing between models include setting, the number of patients in their respective case-loads, the use of outcome measures and the source of the referrals. Beyond the primary function of the management of behaviour that challenges, ISTs also encompass facilitation of discharge of in-patients with mental ill health, assessment and treatment of children with intellectual disability, autism diagnostic assessments and support of those with forensic histories.

In contrast, the national plan for community services for people with intellectual disability^26^ describes a single holistic service model – while acknowledging variation of provision across England – that includes the following nine areas that address key processes and outcomes essential in the lives of people with intellectual disability.

(a) Good and meaningful everyday life.
(b) Person-centred, planned, proactive and coordinated support.
(c) Choice and control.
(d) Support to live in the community with support from and for their families/carers as well as paid care staff.
(e) Housing choices.
(f) Use of mainstream NHS care.
(g) Access to specialist health and social care in the community.
(h) Support to manage antisocial behaviour or police contact.
(i) Access of high-quality in-patient care when needed.

The National Institute for Health and Care Excellence Guideline 11^28^ also recommends intensive community services to provide appropriate crisis response with a focus on preventing admissions for behaviours that challenge.

Just over half of ISTs (52%) reported operating a daily help line (also called duty line) staffed by health professionals with the capacity to respond immediately to crises, and a third of the ISTs provide 24 h and weekend support to adults with intellectual disability and families in distress. However, many respondents across the two models reported that the system can be inflexible, and when an individual is in crisis there is lack of alternatives to admission and of skilled social care providers.

At this time, there is no specific guidance or indication of expected outcomes from ISTs, such as duration of engagement or case-load size beyond reduction in in-patient bed days. Findings from crisis care in adult mental health indicate that compulsory admissions are not reduced in the 2 months following a crisis;^29^ therefore, the role and skills of the staff and of the IST model in the crisis pathway for adults with intellectual disability requires further elucidation.

A study by Firn *et al*^30^ reporting on a 4-year observational study of 112 patients who were mentally ill without intellectual disability, showed that replacing specialist assertive outreach teams with ‘reinforced’ community mental health teams may maintain patients in crisis out of hospital and is well tolerated by patients. Although at this stage we cannot comment on which model is associated with better clinical and cost outcomes, understanding the provision and aspirations of ISTs for people with intellectual disability and behaviours that challenge will provide useful background to service development and care quality improvements.

The IST scope, as we have found, is not as uniform as that of adult mental health crisis resolution teams for a host of reasons including the high heterogeneity of people with intellectual disability, and the significant mental and physical health multimorbidity.

We are now carrying out the second phase of the project, recruiting participants receiving treatment by ISTs in the two models with the view of comparing clinical and cost outcomes and exploring critical components of effective IST models. Further, we are investigating the broader context of the IST and will add to effectiveness and cost data with qualitative interviews of stakeholders within the IST pathway. Those aspects will be pivotal in the wider dissemination and implementation strategy that will be required to ensure that localities heed the findings and set outcomes for the ISTs.

### Strengths and limitations

The study describes a comprehensive survey of ISTs for adults with intellectual disability and/or autism and behaviours that challenge in England and obtained a high completion rate (91%). It expands on recent data of intensive services produced by the NHS Benchmarking Network Learning Disabilities (NHSBNLD)^17^ contributing to an invaluable insight into IST eligibility criteria, staff mix, interventions and other domains considered important to the functions of an IST. The study also has limitations, including being subject to possible respondent social desirability bias. We may have omitted other elements of functions relating to ISTs that could be of importance but not captured in the survey. For example, we did not ask specifically about integration with other local mental health services, gatekeeping role, information sharing or record keeping. Finally, the teams surveyed were those identified during screening and potentially, we may have over/under-ascertained ISTs and therefore the typology may lack precision. As a result of the data available we were only able to include six variables in the cluster analysis. We argue that the resulting typology has an empirical basis and clinical face validity based on clinician experience of such services. However, it has no theoretical basis on literature relating to intellectual disability but has largely adopted functions and indicators from existing literature on mental health crisis care. This may account, to an extent, for both the substantial overlap as well as the variation between clusters on some of the variables.

### Results in context

Davison *et al*^11^ conducted the most recent survey of ‘peripatetic teams’ for people with intellectual disability across the lifespan in England and Scotland. The authors defined a peripatetic service as one that had two or more members of staff; focused on addressing the behavioural needs of the individual in addition to the services that individuals with intellectual disability received on a day-to-day basis. The survey is limited by the response rate of less than 50% (20/46) and did not attempt to develop a typology of ISTs. In response to the transforming care programme recommendations, NHS England funded six services^14^ in England to act as fast-track sites in reducing admissions mainly by enhancing their community services. Given the diversity of the services and the care environments, it is doubtful whether a replicable model of care could emerge without further systematic evaluation of ISTs.

An earlier survey carried out in 1993^31^ found that peripatetic teams employed in excess of 450 staff at a cost of £10 m treating about 2000 individuals. Ayres & Roy^13^ reported that the outreach team they set up costed £490 000 in 2008/2009 prices and employed 11 staff, although FTEs are not reported and the team received additional clinical input by psychiatrists, psychologists and other professionals. Limited existing evidence indicates that ISTs may be clinically effective and possibly cost neutral,^12,32^ however, over time, IST models have been adopted and then decommissioned as new local or national funding priorities arise. Without specific performance indicators it may be difficult to maintain ISTs over time, therefore, it is paramount that further evidence is gathered about their impact on patient well-being that addresses methodological shortcomings of previous research.

### Implications for practice

IST respondents mentioned a number of challenges and priorities, such as extending the opening hours to provide a genuine crisis service. This is a standard part of service models for mental health crisis resolution teams^22^ that in many areas in England provide joint care to patients with mild-to-moderate intellectual disability and mental ill health. It is uncertain, whether extended hours would be sufficiently utilised to cover staffing and other costs without accurate information about numbers of individuals with more severe intellectual disability and behaviours that challenge, who experience crises out of hours and who cannot access mainstream care. Introducing a wider scope for ISTs may also be counterproductive especially if clinical contacts are intervention-poor because of a national shortage of specialists.

We found that London was the area with the smallest number of ISTs. This may be related to the particular mental health service configurations in large urban centres, as it is more likely that people with intellectual disability can access other services such as home treatment teams and psychiatric liaison teams at accident and emergency departments. Consequently, there may be less demand for a highly specialist IST out of hours. However, this approach also requires training of adult mental health service staff and co-working with CIDS to manage emergencies effectively including the adoption of reasonable adjustments.^33^

Despite the proliferation of good practice examples and stated need for intensive care in the community,^34,35^ there is little demonstrable model fidelity and what may be acceptable adaptations across multiple localities. The variation in practice is undoubtedly a significant challenge that supports the need for further evidence on the clinical and cost-effectiveness of ISTs in order to underpin and enhance this important policy drive for patient benefit.

## Data Availability

De-anonymised data included in the survey are available from the authors on request and will be subject to negotiations.
